# Histone deacetylase 1 is required for the development of the zebrafish inner ear

**DOI:** 10.1038/srep16535

**Published:** 2016-02-01

**Authors:** Yingzi He, Dongmei Tang, Wenyan Li, Renjie Chai, Huawei Li

**Affiliations:** 1 Otorhinolaryngology Department of Affiliated Eye and ENT Hospital, State Key Laboratory of Medical Neurobiology, Fudan University, Shanghai, 200031, P.R. China; 2 Key Laboratory of Hearing Medicine of National Health and Family Planning Commission, Shanghai, P.R. China; 3 Institutes of Biomedical Sciences, Fudan University, Shanghai, 200032, P.R. China; 4 Co-innovation Center of Neuroregeneration, Key Laboratory for Developmental Genes and Human Disease, Institute of Life Sciences, Southeast University, Nanjing, Jiangsu, 210096, P.R. China

## Abstract

Histone deacetylase 1 (HDAC1) has been reported to be important for multiple aspects of normal embryonic development, but little is known about its function in the development of mechanosensory organs. Here, we first confirmed that HDAC1 is expressed in the developing otic vesicles of zebrafish by whole-mount *in situ* hybridization. Knockdown of HDAC1 using antisense morpholino oligonucleotides in zebrafish embryos induced smaller otic vesicles, abnormal otoliths, malformed or absent semicircular canals, and fewer sensory hair cells. HDAC1 loss of function also caused attenuated expression of a subset of key genes required for otic vesicle formation during development. Morpholino-mediated knockdown of HDAC1 resulted in decreased expression of members of the Fgf family in the otic vesicles, suggesting that HDAC1 is involved in the development of the inner ear through regulation of Fgf signaling pathways. Taken together, our results indicate that HDAC1 plays an important role in otic vesicle formation.

The vertebrate inner ear is a conserved and complex sensory organ that develops from the otic vesicle and consists of two sensory organs, the auditory apparatus (the cochlea in mammals) and the vestibular apparatus^1^. The incomplete or aberrant development of the auditory organs or damage to the fully developed organs can cause hearing loss. The zebrafish (*Danio rerio*) has become an attractive model organism for the study of the developmental and regulatory mechanisms of hearing and vestibular disorders because it has features that are closely related to the auditory systems of higher vertebrates^2^. During development, the initial steps in otic placode induction are highly complex, and combinations of factors appear to be involved in this process. The family members of the fibroblast growth factors (Fgfs) have been reported to be involved in otic induction. In particular, two ligands of the Fgf signaling pathway, *fgf3* and *fgf8*, are expressed in the hindbrain and cooperate to induce otic development in zebrafish^3^,^4^,^5^. The acerebellar mutation in *fgf8* results in smaller otic vesicles, which have abnormal otoliths and semicircular canals^3^. Knockdown of *fgf3* by injection of morpholino oligomers disrupts otic induction in a similar manner as acerebellar mutation of *fgf8*. Moreover, loss of both *fgf8* and *fgf3* causes severe reduction or total loss of otic tissues^3^,^4^,^5^. A number of Fgf-signaling downstream target genes have been identified as important in otic formation and early ear fate decision. For example, *Pax* genes are the main effectors downstream of Fgf signaling and are involved in otic specification in zebrafish^5^,^6^.

Covalent chromatin modifications play essential roles in transcriptional regulation in eukaryotes^7^,^8^. Acetylation and deacetylation are the most widespread modifications of histones and serve as the key modulators of gene transcription and chromatin structure^9^. Histone acetylation and deacetylation are dynamic processes controlled by the activities of two histone-modifying enzymes, the histone acetyltransferases (HATs) and the histone deacetylases (HDACs). HATs are generally associated with transcriptional activation, while HDACs oppose the activity of HATs by removing acetyl groups from histone tails, which results in chromatin compaction and transcriptional repression^10^. The balance between the activities of HATs and HDACs is a critical regulator of cell differentiation, proliferation, and apoptosis and plays an important role in numerous developmental processes and disease states.

The HDAC family consists of highly conserved enzymes, and these can be divided into four classes based on their involvement in different cellular and developmental processes^10^. The vast majority of studies in HDACs have focused on the knockdown or overexpression of HDACs. Aberrant expression of HDACs has been reported in various cancer types^11^, and HDAC inhibitors (HDACis) are currently attracting enormous attention as anticancer drugs because of their ability to inhibit cancer cell proliferation, induce cell-cycle arrest, and cause cell death^12^,^13^. As epigenetic regulators, HDACs also play important roles in embryonic development^14^,^15^. Class I HDACs, for instance, are present in most cell types and have been shown to be required for proper formation of the eye, central nervous system (CNS), craniofacial cartilage, pectoral fin, liver, and exocrine pancreas^16^,^17^,^18^,^19^. Recently, HDACs have been reported to play essential roles in vertebrate heart tube formation by activating Wnt/β-catenin signaling^20^. However the requirements for individual HDACs have not been fully determined.

HDAC1 has a wide expression pattern and is important for multiple aspects of normal embryonic development^10^. Deletion of murine HDAC1 leads to lethality before embryonic day 10.5, and these mice display severe proliferation defects and growth retardation^21^. In zebrafish, HDAC1 activity is required to promote specification of neural progenitors in the developing brain by antagonizing the gene expression of Notch targets^17^,^22^. HDAC1 has also been shown to exert its function in early dorsal–ventral and brain patterning by repressing the expression of canonical Wnt genes and activating the expression of non-canonical Wnt signaling^23^,^24^. Additionally, HDAC1 has a region-specific effect during embryonic development. In the hindbrain, HDAC1 mutations cause a significant reduction in cell proliferation in zebrafish^17^. In contrast, HDAC1 promotes cell cycle exit in the retina and regulates retinal neurogenesis by blocking Wnt and Notch signaling pathways^19^,^22^.

We have previously reported important roles of HDACs in hair cell regeneration in zebrafish lateral line neuromasts and have investigated the expression of Class I and Class II HDACs after HDACi treatment^25^. In the current work, we have used HDACis in zebrafish embryos to show the requirement of HDAC in ear development. We show that HDACi treatment leads to defects in ear development, including abnormal otoliths and fewer sensory hair cells. We also show that HDAC1 is expressed in the developing inner ear and that knockdown of HDAC1 results in severe malformation of the otolith and semicircular canal during ear morphogenesis. Reduction of HDAC1 activity also affected hair cell differentiation in the inner ear. Finally, we report the association of HDAC1 activity with cell proliferation, cell survival, and the expression of hearing-associated genes. Taken together, these results indicate that the chromatin modification factor HDAC1 might be an important regulator of the formation and maintenance of auditory organs in zebrafish.

## Results

### HDAC inhibition by Trichostatin A (TSA) or valproic acid (VPA) induces otic malformations during embryogenesis

**Figure 1:**
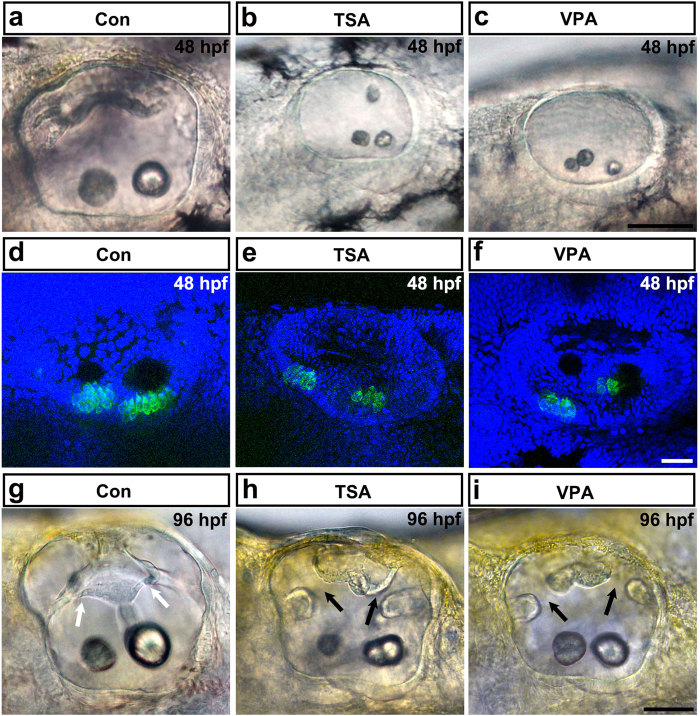
HDAC inhibitor treatment induces otic abnormalities and reduces the number of hair cells in the inner ear. (**a–c**) The morphology of the otoliths in control embryos and in embryos treated with TSA or VPA from 12 hpf onwards. HDAC inhibitor treatment caused morphant otolith defects at 48 hpf of development. **(d–f)** Images of hair cells expressing GFP in control, TSA-treated, and VPA-treated transgenic zebrafish at 48 hpf. Hair cells were detected by *brn3c*:gfp expression. HDAC inhibitor treatment reduced hair cell numbers in the inner ear. **(g–i)** The morphology of the semicircular canals in control (**g**), TSA-treated (**h**), and VPA-treated (**i**) zebrafish. HDAC inhibitor treatment induced aberrant semicircular canal formation. White arrows mark fused pillars, and black arrows mark unfused projections. All images are lateral views with the anterior to the left and the dorsal side up. Scale bars are 50 μm (**a**–**c**,**g**–**i**) and 20 μm (**d**–**f**).

To investigate the function of HDAC activity during inner ear development, zebrafish embryos were continuously treated from 12 hours post fertilization (hpf) onwards with 0.1 μM TSA or 100 μM VPA. At 48 hpf, we found that about 58% (98/169) of the TSA-treated and 42% (43/103) of the VPA-treated embryos exhibited a variety of abnormal otolith morphologies, including multiplication, aggregation, or ectopic location (Fig. 1a–c). In addition, we observed that hair cell numbers in the maculae of HDACi-treated embryos were reduced compared with control embryos at 48 hpf (Fig. 1d–f and Supplemental Figure 1a and e, *p* < 0.05, ANOVA). In order to determine if the timing of TSA or VPA action corresponds to that of hair cell differentiation in zebrafish, two series of experiments were added. First, TSA or VPA pulse treatments were started at 12 hpf and continued until 19 hpf, a time frame covering the main steps of otic placode development. The number of hair cells was examined at 48 hpf. In a second series of experiments, treatment started from 19 hpf onwards until 48 hpf. When TSA or VPA was present from 19 to 48 hpf, embryos had fewer hair cells compared to the controls (Supplemental Figure 1b,d,e, *p* < 0.05, ANOVA). However, transient HDACi treatment from 12 hpf to 19 hpf did not lead to hair cell defects (Supplemental Figure 1c, ANOVA). The embryos that were continuously treated with 0.1 μM TSA or 100 μM VPA also exhibited semicircular canal defects at 96 hpf. As shown in Fig. 1g–i, about 63% (68/108) of the TSA-treated and 37% (42/114) of the VPA-treated embryos showed malformed protrusions of the semicircular canals. These results provided further evidence that HDAC activity is important for proper inner ear development. Because TSA and VPA can efficiently inhibit many HDACs, especially HDAC1, our attention was focused on HDAC1.

### Expression pattern of *hdac1* during zebrafish organogenesis

**Figure 2:**
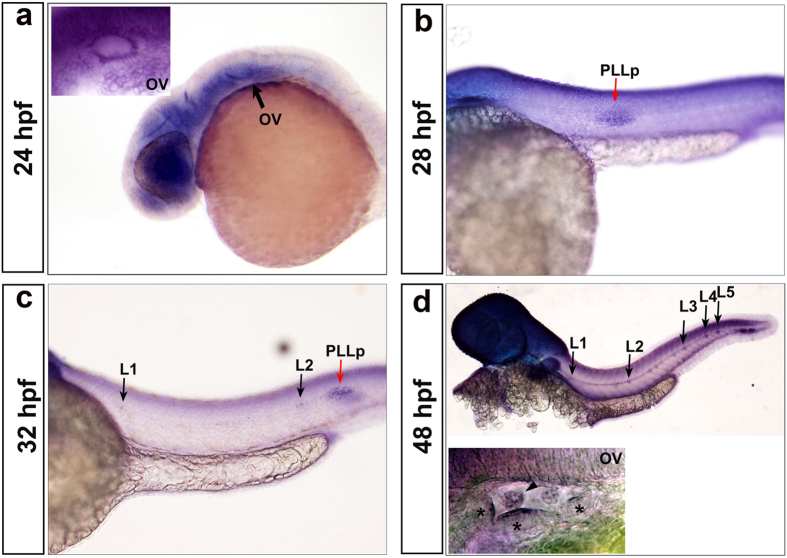
Zebrafish *HDAC1* expression pattern revealed by *in situ* hybridization. **(a–d)** Zebrafish *hdac1* is broadly expressed in the otic vesicle ((**a**,**d**) OV; indicated by black arrows at 24 hpf and 48 hpf), the posterior lateral line primordium (PLLp) ((**b**,**c**); indicated by red arrows at 28 hpf and 32 hpf), and the deposited neuromasts ((**c**,**d**) indicated by black arrows at 32 hpf and 48 hpf). The arrowhead shows protrusions of the semicircular canals, and the asterisks show the three cristae.

Previous studies reported that *hdac1* is ubiquitously expressed in early zebrafish embryos^10^ and is required for the differentiation of the retina^19^,^22^, melanophores^26^, and motor neurons in the CNS^17^ as well as in heart development^27^. Here, we focused on the role of HDAC1 during zebrafish sensory organ development. Whole-mount *in situ* hybridization data showed that *hdac1* expression was mainly concentrated in the encephalic region, eye, and otic vesicle at 24 hpf (Fig. 2a). *Hdac1* was also expressed in the migrating posterior lateral line primordium and the deposited neuromasts, and HDAC1 levels were maintained throughout their development (Fig. 2b,c). At 48 hpf, *hdac1* was detected in the posterior lateral line neuromasts and remained highly expressed in the brain, including the inner ear (Fig. 2d). Our results are consistent with observations by Cunliffe (2004)^17^ and suggest that HDAC1 might be correlated with the development of the inner ear.

### Suppression of HDAC1 results in severe otic vesicle defects

**Figure 3:**
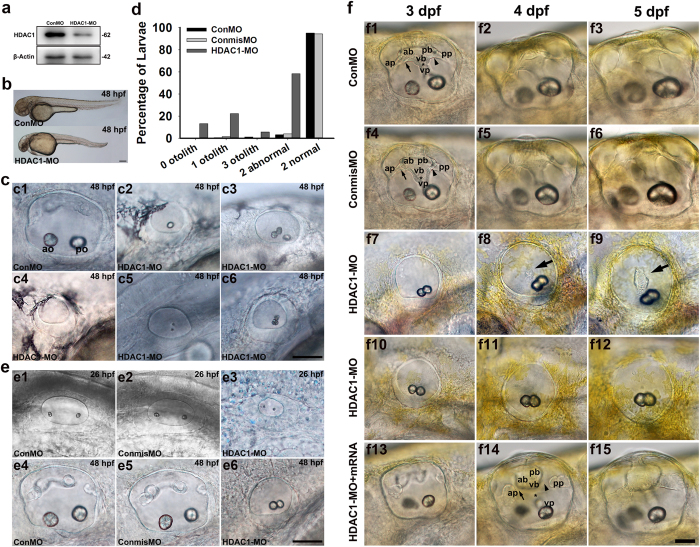
Phenotypes of HDAC1 knockdown by antisense morpholino oligonucleotides. **(a)** The HDAC1 protein level in HDAC1-MO-injected embryos was significantly lower than control embryos at 24 hpf. **(b)** Gross phenotypic morphology of control and HDAC1 morphant embryos. The HDAC1 morphant at 48 hpf shows morphological abnormalities and abnormal otic vesicles. **(c)** Morphological development of the inner ear requires HDAC1. **(c1)** Control larvae (48 hpf) have two normal otoliths. The positions of the anterior otolith (ao) and posterior otolith (po) are indicated. Based on the otolith phenotypes, zebrafish HDAC1 morphants were classified into the following five categories: **(c2)** the one otolith group; **(c3)** the multiple otolith group; **(c4)** the absent otolith group; **(c5)** the two abnormal otolith group; and **(c6)** the two normal otolith group. **(d)** Percentages of embryos in each category (n = 321 for ConMO n = 157 for ConmisMO, and n = 211 for HDAC1 morphants). **(e)** The overall morphology of otic vesicles in control embryos (ConMO and ConmisMO) and HDAC1 morphants at 26 hpf **(e1–e3)** and 48 hpf **(e4–e6)**. **(f)** Zebrafish semicircular canal phenotypes in controls (ConMO and ConmisMO), HDAC1 morphants, and HDAC1-MO + HDAC1mRNA co-injected embryos at 3 dpf, 4 dpf, and 5 dpf. Arrows show the junction of the anterior protrusion (ap) and the anterior bulge (ab). Arrowheads show the junction of the posterior bulge (pb) and posterior protrusion (pp). Asterisks show the junction between the ventral bulge (vb) and ventral protrusion (vp). Arrows in f8 and f9 mark ventral projections. The arrow, arrowhead, and asterisk in f14 mark unfused projections. All images are lateral views with the anterior to the left and the dorsal side up. Scale bars are 200 μm (**b**) and 50 μm (**c**,**e**,**f**).

The HDAC1 gene is expressed in the otic vesicle during zebrafish development. To investigate the role of HDAC1, we used an antisense morpholino oligonucleotide (MO) injected into 1–2-cell stage embryos to suppress translation of HDAC1 protein. The efficiency of the morpholino was confirmed by immunoblot detection (Fig. 3a). In contrast to embryos injected with a control morpholino (ConMO), injection of HDAC1 MO caused obvious defects in the gross morphology of 48-hpf zebrafish such as reduced total body length and cardiac edema (Fig. 3b). The reduction in HDAC1 function also resulted in apparent otic defects. Compared to the embryos injected with ConMO, the HDAC1 morphant embryos displayed significant malformations of otoliths (Fig. 3c1–c6). Otolith defects were observed in almost all of the HDAC1 morphants (>99%) but only in a minority (<5%) of control embryos. We categorized morphants into the following five groups according to otolith number: the absent otolith group; the one otolith group; the two normal otolith group, which had otoliths of comparable size to the wild-type embryos; the two abnormal otolith group; and the multiple otolith group (Fig. 3c–d). For zebrafish with two abnormal otoliths, the otoliths were often visibly smaller and/or in the form of ectopic or fused otoliths.

The absent or one otolith phenotype might be mainly due to the failure of otolith seeding. For HDAC1 morphants with fused otoliths, otoliths were formed but the two otolith precursors might have been seeded too close to each other such that the two otoliths would eventually connect and fuse to become a single otolith. To confirm our hypothesis that HDAC1 is involved in otolith formation, we compared the morphologies of the otic vesicles and the numbers of otoliths between control and HDAC1 morphants early in development. At 26 hpf, the overall morphology of the otic vesicles appeared to be normal in the morphants, but the overall size was reduced (59% compared to controls, n = 40, Fig. 3e1,e3). However, in HDAC1 morphants, fused otoliths located at the position of the saccular maculae were frequently observed, and the size of the otic vesicles was significantly smaller at 48 hpf (68% compared to controls, n = 76) (Fig. 3e4,e6). In addition to the otolith phenotypes, the development of semicircular canals was also abnormal throughout embryonic development. By 3 dpf, HDAC1 morphants showed aberrant semicircular canal formation with absent protrusions of semicircular canals (Fig. 3f) that is consistent with the absence of semicircular canal projections reported by Cunliffe in HDAC1 mutants, and this suggests that HDAC1 function is required for the formation of semicircular canals^17^. This is not likely due to developmental delay because fusion failure was found in HDAC1 morphants at much later stages (4 and 5 dpf) and because the defects were seen in all three canals. Additionally, our analysis showed that the embryos injected with a 5-bp mismatch MO (ConmisMO) at the same concentration did not produce any of the otic defects observed in HDAC1 morphants (Fig. 3d–f). In order to verify that the semicircular canal defect observed in embryos injected with HDAC1 MO was specifically due to disruption of HDAC1 function, we injected zebrafish HDAC1 mRNA in combination with HDAC1 MO. In this assay, we observed that the majority of the co-injected embryos showed outgrowth of semicircular canals (Fig. 3f15) indicating a partial rescue of the HDAC1 MO phenotype. Taken together, these results suggested that reduction of HDAC1 activity leads to malformations of the otoliths and semicircular canals in the inner ear.

### Suppression of HDAC1 results in fewer hair cells

**Figure 4:**
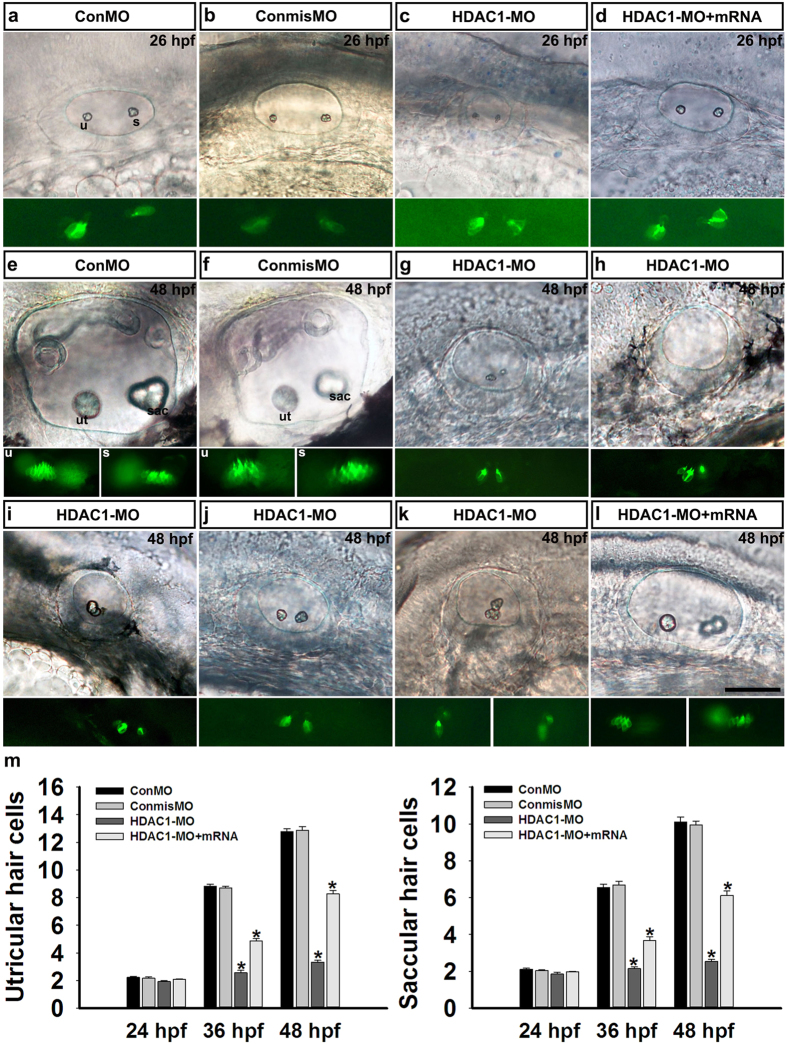
Hair cells in the inner ear. HDAC1 morphants exhibited a normal number of hair cells at 26 hpf but showed significantly fewer hair cells at later time points. **(a–d)** *Brn3c*:gfp expression in the inner ear of a control MO-injected embryo (ConMO) **(a)**, a mismatch MO-injected control embryo (ConmisMO) **(b)**, an HDAC1 morphant **(c)**, and an HDAC1-MO + HDAC1mRNA co-injected embryo **(d)** at 26 hpf. **(e–l)** *Brn3c*:gfp expression in the utricle and saccule of a control MO-injected embryo (ConMO) **(e)**, a mismatch MO-injected control embryo (ConmisMO) **(f)**, HDAC1 morphants **(g–k)**, and an HDAC1-MO + HDAC1mRNA co-injected embryo **(l)** at 48 hpf. All images show lateral views with the anterior to the left and the dorsal side to the top. Positions of the utricular (ut) and saccular (sac) maculae are indicated. **(m)** Quantification of the numbers of hair cells in the inner ear for each experimental condition at the indicated stages. Data are shown as mean ± SEM (n = 25–45). *p < 0.05. Scale bars, 50 μm.

To determine whether knockdown of HDAC1 perturbs hair cell development in the inner ear, we injected HDAC1 MO into transgenic Tg(*brn3c*:gfp) zebrafish that express GFP in differentiated hair cells^28^. We first examined the formation of early hair cells (named tether cells) that initially form in pairs and are required for otolith localization^29^. At 26 hpf, HDAC1 morphants were similar to control embryos, which had a pair of tether cells at both the anterior and posterior ends of otic vesicle (Fig. 4a,c), but occasionally tether cells in HDAC1 morphants appeared to be closer to each other and located on the inside of the otic placode rather than in their correct location. These will eventually fuse at the location of the saccular macula to form a single sensory patch, and this was accompanied by fusion of the two otoliths at 48 hpf (Fig. 4k). Compared to controls, there were significantly fewer later-forming hair cells in all HDAC1 morphants (*p* < 0.05, ANOVA) (Fig. 4m). Additionally, our analysis showed that the embryos injected with ConmisMO had no defects in hair cell development (Fig. 4b,f, and m, ANOVA). Because otic expression *of atoh1a* began at 14 hpf in two domains in the otic placode, which marked the primordia of the utricular and saccular sensory epithelia, we next examined expression of *atoh1a* in embryos injected with HDAC1 MO. In HDAC1 morphants, *atoh1a* was expressed normally at 14 hpf (Supplemental Figure 2). Together, these results indicated that the absence of HDAC1 has no apparent effect on otolith seeding. However, HDAC1 might be an important regulator in later-forming hair cell formation, which was also confirmed by the effect of HDAC inhibition on hair cell development in the inner ear of zebrafish (Supplemental Figure 1). To confirm whether the effects of HDAC1 MO on hair cell development could be rescued, we co-injected HDAC1 MO with HDAC1 mRNA and observed that co-injected embryos produced more GFP-positive later-forming hair cells than HDAC1 morphants (Fig. 4l,m).

### Cell division and cell death are affected by suppression of HDAC1

**Figure 5:**
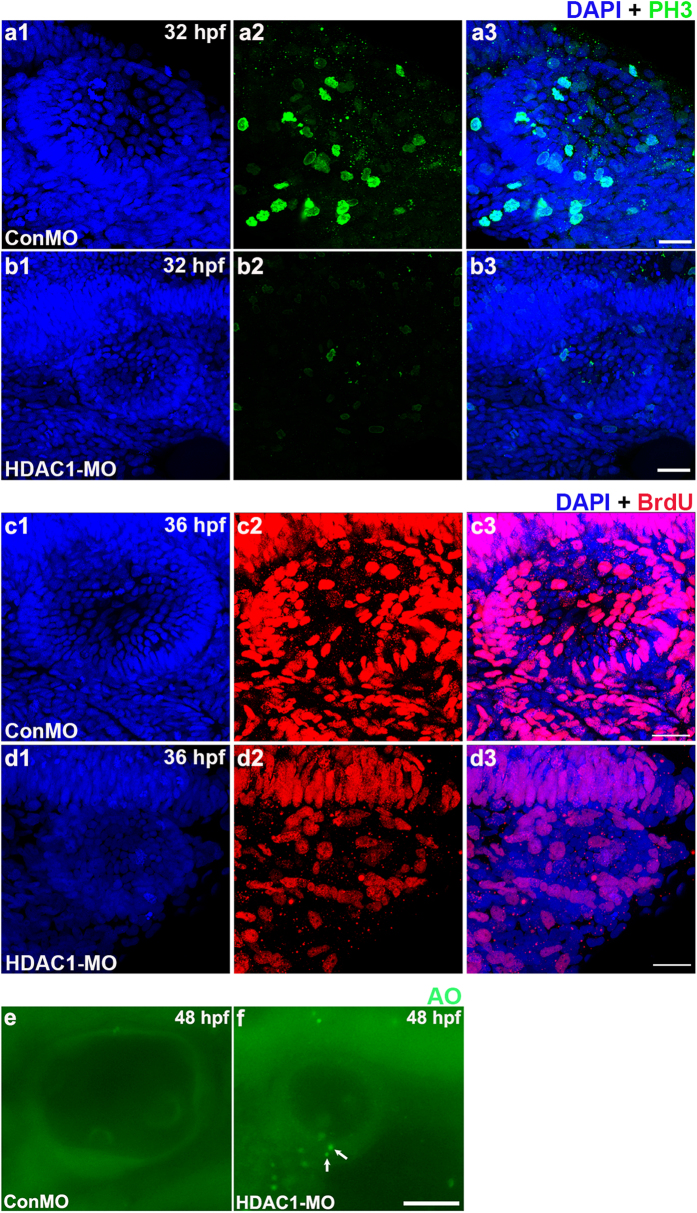
Analysis of cell proliferation and death in the otic vesicle of HDAC1 morphants. (**a**,**b**) Mitotic cells in the 32 hpf otic vesicle of a control embryo **(a)** and an HDAC1 morphant **(b)** were identified by phospho-histone H3 expression, a mitotic marker. **(c,d)** Dividing cells in the otic vesicle of a control embryo **(c)** and an HDAC1 morphant **(d)** at 36 hpf were identified by BrdU staining. HDAC1 morphants often contained decreased numbers of proliferating cells within the sensory epithelia. **(e,f)** AO labeling of dying cells in the otic vesicle of a control embryo **(e)** and an HDAC1 morphant **(f)** at 48 hpf. White arrows indicate AO-positive cells. HDAC1 morphants often contained multiple dying cells within the sensory epithelia. All images show lateral views with the anterior to the left and the dorsal side up. Scale bars, 20 μm (**a**–**d**), Scale bars, 50 μm (**e**,**f**).

It has been previously observed that HDAC1 plays a role in regulating cell proliferation during mouse and zebrafish development^17^,^19^,^21^, and we have shown that disruption of HDAC1 perturbs otic development and reduces the number of hair cells. To test whether the reduced hair cell number was due to changes in cell proliferation, we used the phospho-histone H3 (PH3) antibody, which labels the cells in M phase, to mark mitotic cells, and observed that the number of PH3-positive cells was significantly reduced in HDAC1 morphant otic vesicles at 32 hpf compared with controls (22 ± 10.5 and 4.4 ± 2.3 PH3-positive cells in ConMO (n = 12) and HDAC1 MO (n = 16) otic vesicles, respectively; *p* < 0.001, Student t-test) (Fig. 5a,b). We used BrdU uptake to determine the numbers of proliferating cells in the otic vesicles at 36 hpf and found an apparent decrease in BrdU-positive cells in HDAC1 morphant otic vesicle compared to controls (Fig. 5c,d). Interestingly, these proliferating cells seem to be mainly localized to the anterior and posterior otic epithelium in the inner ear of control embryos, whereas there were very few proliferating cells in HDAC1 morphants, which is in agreement with the smaller inner ear and reduced number of hair cells observed in HDAC1 morphants.

To test whether increased cell death of placodal cells also contributes to the reduction of otic vesicle size in HDAC1 morphants, we used acridine orange (AO) vital dye to examine the dying cells. At 48 hpf, we found significantly more AO-positive cells in HDAC1 morphants than in control otic vesicles (1.6 and 4.7 AO-positive cells per ear in controls (n = 37) and HDAC1 morphants (n = 40), respectively; p < 0.001, Student t-test) (Fig. 5e–f). The majority of AO-positive cells (56%) were seen within the developing maculae of HDAC1 morphants. These results indicated that cell death is nearly undetectable in sensory epithelial cells of controls, but is common in HDAC1 morphants, and this confirmed that HDAC1 directly or indirectly influences hair cell survival. Taken together, these data show HDAC1 is required in regulating cell proliferation and survival within the inner ear.

### Effect of HDAC1 knockdown on the expression of otic markers

**Figure 6:**
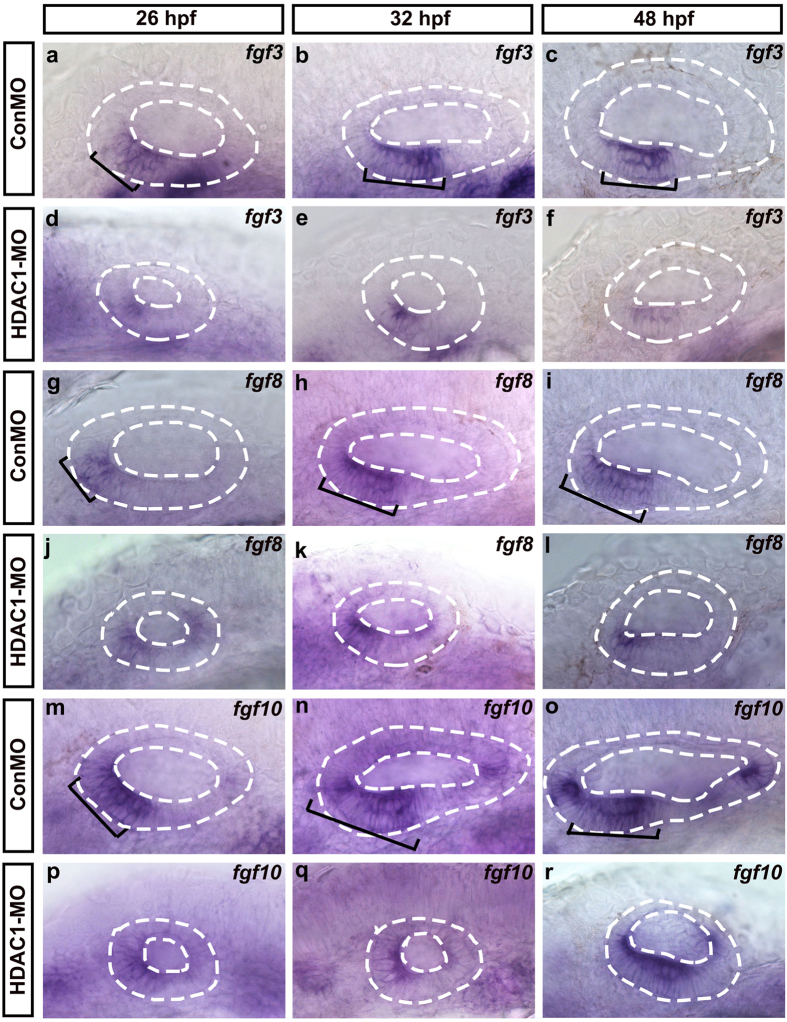
The effect of HDAC1 knockdown on the expression of *fgf3/8/10* in the developing inner ear. Whole-mount *in situ* hybridizations with *fgf3* **(a–f)**, *fgf8* **(g–l)**, and *fgf10* **(m–r)** probes in control embryos **(a–c,g–i,m–o)** and HDAC1 MO-injected embryos **(d–f,j–l,p–r)** at different developmental stages (as indicated). Black brackets indicate the localization of *fgf3*-expressing **(a**–**c)**, *fgf8*-expressing **(g–i),** and *fgf10*-expressing **(m–o)** cells. Embryos injected with HDAC1 morpholino show reduction in levels of *fgf3* **(d–f)**, *fgf8* **(j–l),** and *fgf10* **(p–r)** expression. The otic vesicles are outlined by dashed lines. All images show lateral views with the anterior to the left and the dorsal side up.

To determine whether or not HDAC1 is required at the earliest stages of otic placode development, we examined expression of three early placode markers—*pax2a, cldna*, and *eya1*—in embryos injected with HDAC1 MO at 14 hpf. We found that HDAC1 MO injection did not affect the expression of otic *pax2a, cldna*, or *eya1* (Supplemental Figure 3), and the overall size of the otic placode was normal in the morphants. This suggests that the absence or reduced size of otic vesicles in HDAC1 morphants might not be due to defects in the earliest stages of otic placode induction. To better understand the role of HDAC1 in otic development, we performed an *in situ* hybridization to examine the expression of several genes known to regulate otic vesicle specification and patterning. Mutational studies in zebrafish have suggested an important role for Fgf signaling in otic induction and vesicle patterning^5^,^30^, and in the current work we first tested whether reduction of HDAC1 activity affects the expression of Fgf genes. We found that the zebrafish *fgf3, fgf8*, and *fgf10* genes were expressed normally in the ventroanterior maculae of the otic vesicles in control embryos at 26 hpf, 32 hpf, and 48 hpf, but in morphants the reduction of HDAC1 activity appears to have reduced the expression of these genes during otic development (Fig. 6). These results suggested that HDAC1 might be required to maintain the expression of Fgf ligands in the developing anterior otic vesicle.

**Figure 7:**
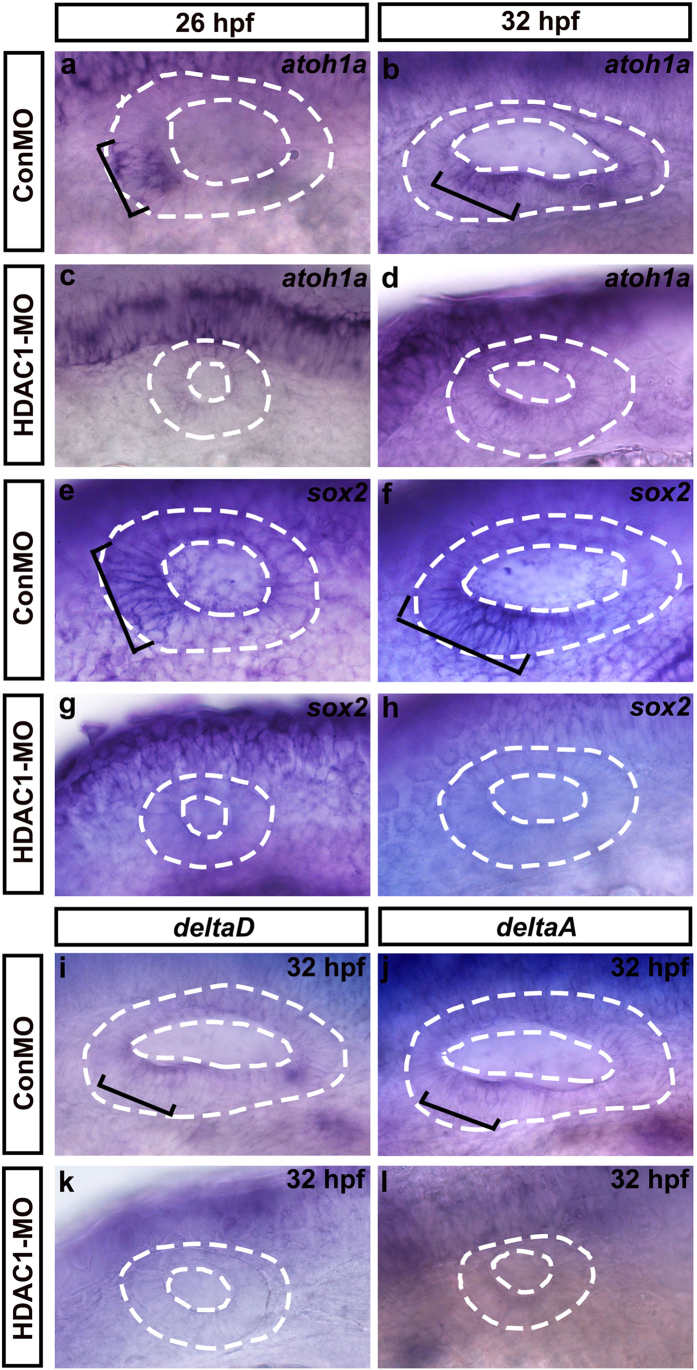
Whole-mount *in situ* hybridizations to *atoh1a, sox2, deltaD*, and *deltaA*. Lateral views of control (ConMO) and HDAC1 morphant otic vesicles at 26 hpf and 32 hpf. Black brackets indicate the regions of *atoh1a*-expressing **(a,b)** and *sox2*-expressing **(e**,**f)** cells as appropriate. Embryos injected with HDAC1 morpholino show a reduction in levels of *atoh1a* **(c**,**d**) and *sox2* **(g,h)** expression. (**i–l**) Whole-mount *in situ* hybridizations to *deltaD* and *deltaA*. Lateral views of control (ConMO) and HDAC1 morphant otic vesicles at 32 hpf. Black brackets indicate the localization of *deltaD*-expressing **(i)** and *deltaA*-expressing **(j)** cells. Loss of function of HDAC1 reduced the expression of *deltaD* **(k)** and *deltaA* **(l)**. Otic vesicles are outlined by dashed lines. All images show lateral views with the anterior to the left and the dorsal side up.

*Math1* (mouse atonal homolog 1), a bHLH proneural gene, has been shown to be required for hair-cell specification in mice^31^. At 26 hpf, *atoh1a* (the zebrafish homolog of *math1*) was expressed in the anterior ventral otic domain that contains precursors of both hair cells and supporting cells (Fig. 7a). When HDAC1 was knocked down, anterior expression of *atoh1a* was significantly reduced compared to control otic vesicles (Fig. 7c). At 32 hpf, *atoh1a* expression was still visible in the sensory maculae in controls (Fig. 7b), but in HDAC1 morphants *atoh1a* expression was diminished and marked only a few cells in the anterior patch (Fig. 7d). We next examined the expression of *sox2*, which is normally co-expressed with *atoh1a* and marks both hair cells and supporting cells in the developing sensory epithelia. At 26 hpf and 32 hpf, the expression of *sox2* in controls was intense along the ventral otic vesicle floor with elevated expression in the macular domains, from which the utricular and saccular maculae will develop (Fig. 7e,f), and there was a significant reduction in the expression of *sox2* in HDAC1 morphants at these stages (Fig. 7g,h).

**Figure 8:**
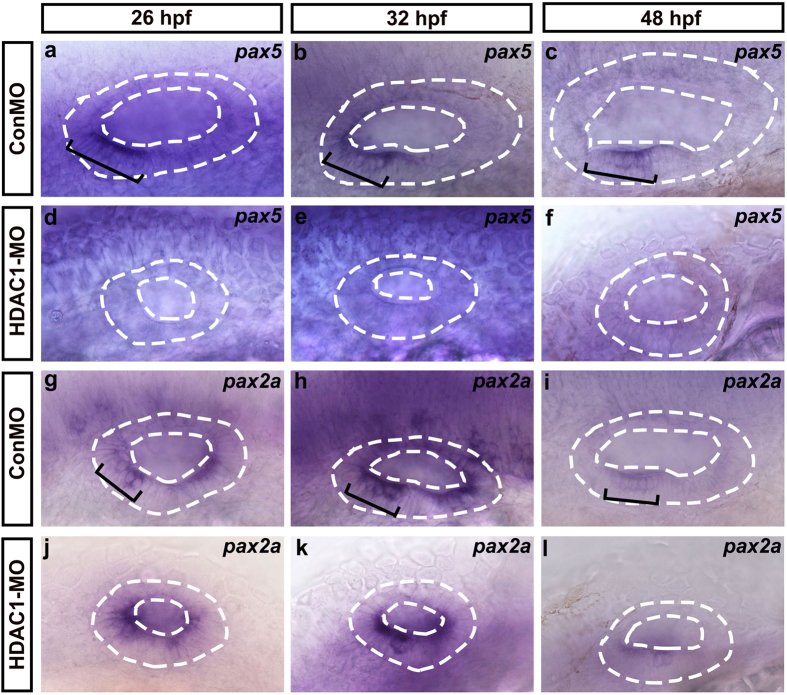
Whole-mount *in situ* hybridizations to *pax5* and *pax2a*. **(a–l)** Lateral views of control (ConMO) and HDAC1 morphant otic vesicles at 26 hpf, 32 hpf, and 48 hpf. Black brackets indicate the localization of *pax5*-expressing **(a–c)** and *pax2a*-expressing **(g–i)** cells as appropriate. Embryos injected with HDAC1 morpholino show reductions in the levels of *pax5* **(d–f)** and *pax2a* **(j–l)** expression. The otic vesicles are outlined by dashed lines. All images show lateral views with the anterior to the left and the dorsal side up.

Notch pathway genes *deltaD* and *deltaA* are the earliest targets of *atoh1* in the zebrafish inner ear, and they eventually become restricted to emerging hair cells^32^. Therefore, the defects in the otic maculae in HDAC1 morphants prompted us to examine the expression patterns of *deltaD* and *deltaA*. We found that both *deltaD* and *deltaA* expression in the ventromedial wall of the otic vesicle was severely reduced or ablated at 32 hpf in HDAC1 morphants (Fig. 7i–l). We next examined the expression of Pax-family genes, which are required for development and maintenance of hair cells within the sensory patches in the otic vesicle^33^. Knocking down HDAC1 diminished *pax5* expression, which is normally expressed in the anterior domain of the otic vesicle at 26 hpf and is restricted to the anterior-medial domain at 48 hpf (Fig. 8a–f). Down-regulation of HDAC1 did not obviously diminish *pax2a* expression (which is a ventromedial marker of the otic vesicle) at 26 hpf (Fig. 8g,j), but at 32 hpf and 48 hpf *pax2a* expression in the anterior macula of the HDAC1 morphants was significantly reduced compared to controls (Fig. 8h–i,k–l). These data showed that full expression of *pax5* and *pax2a* requires HDAC1 function.

## Discussion

Epigenetic chromatin remodeling events are essential for a wide range of cellular processes and disease states^34^. By altering chromatin structure, HDACs play important roles in cell growth arrest and subsequent differentiation. HDAC1 is a class I enzyme that has been shown to be ubiquitously expressed in early zebrafish embryos. HDAC1 has been shown to be required in the differentiation of the heart, eyes, neurons, oligodendrocytes, pectoral fin, and ear in zebrafish^17^,^18^,^19^,^22^. A recent study using antisense morpholino-mediated knockdown of HDAC1 in zebrafish also suggested that HDAC1 is involved in oligodendrocyte development by regulating Wnt signaling^35^. Class I HDACs are highly expressed in the developing brain and otic vesicles in both chicken and mouse embryos^36^, but the function of HDAC1 in inner ear organogenesis is less well characterized. In this study, consistent with previous observations, the whole-mount *in situ* hybridization results clearly demonstrated the expression of HDAC1 in the developing inner ear of the zebrafish, suggesting the potential role of HDAC1 in the development of auditory organs.

We investigated the function of HDAC1 during inner ear development by examining morpholino antisense oligonucleotide knockdown in zebrafish embryos. Placode induction and vesicle cavitation appeared to occur normally, but morphogenesis of the otic vesicle was affected by HDAC1 deficiency. We observed obvious differences in the phenotypes of the ear between HDAC1 morphants and controls. The significant morphological defects of HDAC1 morphants included impairment of otolith formation, reduced otic vesicle size, and reduced numbers of hair cells in the maculae of the inner ear. Therefore, we proposed that HDAC1 deficiency likely disrupts the biological processes that are critical for the formation of the otic vesicle in the zebrafish.

HDAC1 plays a crucial role in regulating cell proliferation and cell death during development. HDAC1 mutant mouse embryos died at very early stages of development, mainly due to an overall reduction of proliferation that was correlated with a decrease in cyclin-dependent kinase activity and upregulation of the cyclin-dependent-kinase inhibitors *p21* and *p27*^21^. In zebrafish, HDAC1-deficient embryos pass through gastrulation and survive longer and develop to later embryonic stages than mouse HDAC1 mutants, but HDAC1-deficient embryos also exhibit a severe defect of cell proliferation in the hindbrain^17^,^23^,^24^. Here we found that HDAC1 deficiency reduced cell proliferation and induced cell death in the zebrafish ear, which might contribute to the reduced number of later-forming hair cells. Moreover, the role of HDAC1 activity in cell cycle regulation is not uniform but is tissue specific. HDAC1 stimulates proliferation in many contexts during development, but in the zebrafish retina HDAC1 promotes cell cycle exit, and *cyclin D* and *E* transcripts are up-regulated in the retina when HDAC1 activity is lost^19^. This suggests that HDAC1 has both positive and negative roles in cell cycle regulation depending on the location and the cell types. We found that HDAC1 deficiency led to reduced proliferation over a broader region in the zebrafish inner ear, and this is consistent with previous reports that there is an overall reduction of cell proliferation in zebrafish HDAC1 morphant embryos. Decreases in cell proliferation in the otic vesicles of HDAC1 morphants could also be due to induction of cell death, so we performed a cell death analysis by AO staining. We observed significant differences in the number of AO-positive cells in the otic epithelium of HDAC1 morphants compared to controls, indicating the important role of HDAC1 in cell survival in the otic vesicle of zebrafish. Additionally, we found that the otic defects in HDAC1 morphants can be partially rescued by co-injection of mRNA indicating that it has a redundant function. It is possible that HDAC1 is more globally required for embryogenesis, consistent with the global expression of this gene in zebrafish embryos. Thus, HDAC1 morphants showed severe developmental defects similar to *hdac1* mutants reported earlier^16^,^17^. Although we cannot exclude the possibility that the relationship between otic defects and inherent variability of the antisense MO, the 5-bp mismatch HDAC1 MO (ConmisMO) was injected at the same concentration to better address this problem.

Our results indicate that HDAC1 is essential for otic morphogenesis in zebrafish, but very little is known about the mechanisms of HDAC1 in ear development. Previous zebrafish studies have identified many developmental signaling molecules that are responsible for the formation of the otic vesicle^37^. As an important epigenetic (transcriptional) regulator, HDAC1 deficiency could alter the normal expression of many genes involved in developmental processes. HDACs have been implicated in various developmental signaling events, including homeobox gene repression^38^, Fgf signaling^17^,^39^, Notch signaling^17^, and Wnt signaling^22^,^23^,^24^. Studies in zebrafish reported that hindbrain-derived Fgf family members are involved in the initial induction of the otic placode and that otic-derived Fgf signals are later involved in the formation and patterning of the otic vesicle, and inhibition of Fgf can prevent inner ear development^3^,^5^,^40^,^41^. For example, *fgf3* and *fgf8* are expressed in the hindbrain and have redundant roles in zebrafish otic patterning^3^,^4^,^5^. The zebrafish *fgf3* or *fgf8* mutants have fused otoliths and fewer hair cells in the utricular maculae at 48 hpf^42^,^43^. These results were similar to HDAC1 morphants, which have smaller otic placodes and vesicles as well as fused otoliths, and this prompted us to investigate the temporal and spatial expression patterns of Fgf genes in HDAC1 morphants. At 26 hpf, the expression of *fgf3, fgf8*, and *fgf10* was observed in the ventroanterior quadrant of the ear in HDAC1 morphants, which was similar to controls, but the expression level was greatly reduced compared to controls. At 48 hpf, the expression of *fgf3, fgf8*, and *fgf10* was shifted to the ventral side of the inner ear in control embryos, while in many morphants the expression was significantly decreased in the anterior side of the ear, suggesting that the expression domains were affected by the reduction in HDAC1 activity. However these expression domains were not entirely eliminated, indicating that the differentiation of otic epithelium still occurred to some extent without HDAC1. The requirement for HDAC activity in regulating Fgf signaling has been shown in Xenopus mesoderm induction and in the developing zebrafish^39^,^44^. HDAC1/2 can be recruited to a transcriptional corepressor, RERE, which is implicated in multiple embryonic tissues by modulating the Fgf signaling pathway during embryogenesis, and this imparts tissue specificity to histone deacetylation^44^. Thus it will be interesting to investigate the potential interactions between the Fgf signaling pathway and HDAC1 during zebrafish inner ear development. Interestingly, we found that HDAC1 morphants failed to form semicircular canal pillars at 96 hpf; however, *fgf3* knockdown embryos have normal semicircular canal pillar and crista development, suggesting that other key developmental regulators might cooperate with HDAC1 activity to induce the development of the semicircular canal. Therefore, it will be important to identify the gene targets of HDAC1 regulation and to determine how HDAC1 mediates the different signaling events during otic development and semicircular canal morphogenesis.

In this study, we showed that the formation of the tether cells in early development is normal in HDAC1 morphants. However, HDAC1 deficiency leads to the reduction of later-forming utricular and saccular hair cells in a manner similar to the phenotypes observed in the *atoh1a* morphant^32^. *Atoh1a* is necessary for hair cell development, and it is primarily responsible for specifying later-forming hair cells in the zebrafish inner ear. We examined the expression pattern of *atoh1a* in the HDAC1 morphants by whole-mount *in situ* hybridization and found that HDAC1 deficiency strongly diminished *atoh1a* expression in the developing otic maculae, suggesting that HDAC1 might be required for the normal activation of *atoh1a* gene expression. It has been shown that zebrafish *atoh1a* is regulated by Fgf signaling, and *fgf3* and *fgf8* are upstream activators of *atoh1a* gene expression during the development of the ear^32^. Thus, in the current study, the reduced expression of *atoh1a* in the otic vesicles of HDAC1 morphant embryos is probably due to the loss of Fgf signals. This suggests that appropriate levels of HDAC1 are necessary for regulation of the Fgf signaling pathway and subsequent activation of genes required for hair cell development in the zebrafish inner ear.

A variety of genes are induced and maintained by Fgfs at the otic placode and otic vesicle stages of ear development. For example, the transcription factors *pax2* and *pax5*, which are expressed at specific times, are required for specification and patterning of otic vesicles and play key roles in otic development^33^,^37^. *Pax2* is abundantly expressed at the midbrain–hindbrain boundary and in the hindbrain, the optic stalk, and the otic vesicles^41^,^45^. In zebrafish, loss of *pax2* affects hair cell development but does not hinder otic induction^46^. A recent study showed that HDAC1 and HDAC2 play an important role in the regulation of *pax2*. HDAC inhibition stimulates histone hyperacetylation of *pax2* and significantly reduces its expression during renal development^47^. *Pax5* is initially detected in the anterior of the nascent otic vesicle at a uniformly high level at 24 hpf, but later it is restricted to the utricular macula^6^,^33^. Knockdown of *pax5* during larval development causes vestibular defects and results in the failure to produce the appropriate number of utricular hair cells due to excess apoptosis^6^. In our experiments, *pax2a* and *pax5* were strongly expressed at the anterior end of the otic vesicle in control embryos, but both were severely reduced in HDAC1 morphant embryos. Previous studies reported that the expression pattern of *pax5* in the anterior quarter of the otic vesicle is dependent on Fgf signaling and that *fgf3* serves as an upstream regulator of *pax5*^6^,^33^. In zebrafish, *fgf3* morphants and *fgf3*-null mutants have very similar defects in the ear as the HDAC1 morphants described here, with strong reduction in *pax5* expression in the anterior otic vesicle. These observations indicate that *Pax* gene expression might be regulated by HDAC1 and that this expression requires Fgf signaling during otic vesicle development. Taken together, our analysis showed that the expression of inner ear markers (*fgf3, fgf8, fgf10, atoh1a, sox2 deltaD, deltaA, pax2a,* and *pax5*) are all significantly down-regulated in the otic vesicle of HDAC1 morphants. Although these transcript analysis results support the idea that the events of inner ear development, such as organogenesis and specification, were affected by HDAC1 inhibition, we cannot completely rule out the possibility that the reduced size of the otic vesicle is responsible for the down-regulation of inner ear markers. Microarray analysis will provide important directions for future studies regarding how HDAC1 effects specific genes and developmental processes.

In summary, we found strong HDAC1 expression in the developing otic vesicles of the zebrafish by whole-mount *in situ* hybridization. We found that knockdown of HDAC1 affects the development of the otic vesicle and reduces the number of hair cells in the inner ear. Decreased cell proliferation and increased apoptosis were observed in the HDAC1 morphants, which might lead to the observed reduction in size of the otic vesicles. Finally, we demonstrated the role of HDAC1 in regulating the Fgf pathway in the zebrafish inner ear. The influence of HDAC1 on Fgf signaling might directly or indirectly influence the development of the inner ear in zebrafish. However, the molecular details of this interaction require further investigation. Our findings provide insight into the role of HDAC1 in otic vesicle formation and suggest that maintenance of histone deacetylation at an appropriate level is critical for normal organogenesis.

## Materials and Methods

### Zebrafish strains and maintenance

All zebrafish animal experiments were performed following the institutional guidelines approved by the Institutional Animal Care and Use Committee of Fudan University, Shanghai. The experimental protocols were also approved through the Institutional Animal Care and Use Committee of Fudan University, Shanghai. The methods were performed in accordance with the approved guidelines. Zebrafish were maintained in our facility according to standard procedures. The transgenic line *brn3c*:mGFP was obtained from Professor Zhengyi Chen, our collaborator at Harvard University, while all other embryos were obtained from natural spawning of wild-type adults. The age of embryos and larvae are described as hours post-fertilization (hpf). To prevent pigment formation, embryos older than 48 hpf were treated with 0.003% 1-phenyl-2-thiourea (PTU; Sigma-Aldrich, St Louis, MO, USA) in fish water from 14 hpf onwards.

### Pharmacological treatments

Valproic acid (VPA; Sigma-Aldrich) and Trichostatin A (TSA; Sigma-Aldrich) were dissolved either in water (VPA) or DMSO (TSA) at stock concentrations of 200 mM and 500 μM, respectively, and then diluted to the indicated concentrations in fish water. Embryos were raised up to 12 hpf and then HDAC inhibitors were added to the fish water. Embryos were soaked in VPA- or TSA-containing fish water until an appropriate stage. The fish water was replaced every day with fresh chemicals.

### Morpholino injection and mRNA rescue

A translation-blocking morpholino oligomer (MO) targeting HDAC1 was purchased from Gene Tools, Inc. (Philomatch, Oregon, USA), and was diluted in sterile water at a stock concentration of 1 mM. The sequences were as follows: HDAC1-MO (5 ng)—TTG TTC CTT GAG AAC TCA GCG CCA T, 5-bp mismatch control (ConmisMO)—TTc TTC CTT cAG AAg TCA cCG CgA T^16^, and standard control MO (ConMO) (6 ng)—CCT CTT ACC TCA GTT ACA ATT TAT A. To rescue the HDAC1 MO-injected embryos, HDAC1 5′-capped mRNA was synthesized using the mMACHINE *in-vitro* transcription kit (SP6; Ambion, Austin, TX, USA) according to the manufacturer’s instructions.

### Whole-mount *in situ* hybridization and immunolabeling

Whole-mount *in situ* hybridization was performed according to standard procedures^48^. Digoxigenin-labelled probes were prepared as recommended by the manufacturer (Roche, Mannheim, Germany). Details of the probes used are available on request. PH3 (Abcam, Cambridge, UK) immunostaining was performed using standard staining procedures. Briefly, embryos were fixed for 2 h at room temperature (RT) in 4% paraformaldehyde (PFA). Fixed embryos were blocked using 10% newborn donkey serum and incubated with antibodies overnight at 4 °C. PH3 was used at a 1:1000 dilution. Fluorescently labeled embryos were captured with a Leica confocal fluorescence microscope (TCS SP5; Leica, Wetzlar, Germany). Bright-field images were acquired using a Leica fluorescent microscope (Heerbrugg, Switzerland). Images were processed using Photoshop software (Adobe).

### BrdU incorporation and analysis

BrdU incorporation was performed as described^15^. Briefly, zebrafish embryos were incubated in 10 mM 5-bromo-2-deoxyuridine (BrdU; Sigma-Aldrich) between 35 hpf and 36 hpf at 28.5 °C. BrdU incorporation was detected by immunocytochemistry. Larvae were anesthetized in MS-222 (Sigma-Aldrich) and fixed with 4% PFA for 2 h at RT. The fixed larvae were washed three times in PBS with 0.5% Triton X-100 (PBT-2) and placed into 2N HCl for 0.5 h at 37 °C. Larvae were washed again in PBT-2, and nonspecific binding was blocked with blocking solution for 1 h at RT followed by incubation with mouse monoclonal BrdU antibody (1:200 dilution; Santa Cruz, Dallas, TX, USA. Cat. no. sc-32323) overnight at 4 °C. The next day, the larvae were washed and incubated with secondary antibody for 1 h at 37 °C. Fluorescently labeled embryos were captured with a Leica confocal fluorescence microscope (TCS SP5; Leica). Images were processed using Photoshop software (Adobe).

### Acridine orange staining

Apoptosis in whole zebrafish larvae was observed with the vital dye acridine orange (acridinium chloride hemi-[zinc chloride], AO; Sigma-Aldrich). AO staining was performed as described previously^49^. Briefly, larvae were placed in 2 μg/mL AO in fish water in the dark at 28 °C for 30 minutes. Larvae were then washed with fish water 3 times for 10 minutes each. Images were obtained with confocal microscopy (TCS SP5, Leica) and were processed using Photoshop software (Adobe).

### Western blot analysis

Total protein was isolated with the AllPrep DNA/RNA/Protein Mini Kit (QIAGEN, Hilden, Germany) according to the manufacturer’s instructions. Protein concentrations were measured using a BCA protein kit (Thermo Fisher Scientific, Rockford, IL), and proteins were separated on SDS-polyacrylamide gels and transferred onto PVDF membranes (Immobilon-P; Millipore, Bedford, MA, USA). The membranes were blocked with 5% nonfat dried milk in TBST (50 mM Tris-HCl (pH 7.4), 150 mM NaCl, and 0.1% Tween-20) for 1 h at RT and then blotted overnight with HDAC1 antibody (1:1000 dilution, Cell Signaling Technology Inc., Danvers, MA, USA) or anti-β-actin antibody (1:1000 dilution, Sigma) at 4 °C.

### Statistics

Prior to analysis, all data were first examined for normality and homogeneity of variances by the Shapiro–Wilk test and Levene’s test, respectively. Data were analyzed by either Student’s t-test or analysis of variance (ANOVA) with multiple comparisons using SigmaPlot (version 12.0 for Windows; Systat Software Inc., CA, USA). All data are presented as the mean ± SD. A *p*-value < 0.05 was considered statistically significant, and *p* < 0.001 was considered highly significant.

## Additional Information

**How to cite this article**: He, Y. *et al.* Histone deacetylase 1 is required for the development of the zebrafish inner ear. *Sci. Rep.* **6**, 16535; doi: 10.1038/srep16535 (2016).

## Supplementary Material

Supplementary Information
