# Supplementary material for: Histone deacetylase 1 is required for the development of the zebrafish inner ear
Source: Sci Rep. 2016 Feb 1;6:16535. doi: 10.1038/srep16535 (PMC4735278; doi:10.1038/srep16535)
Supplement: Supplementary Information [file srep16535-s1.pdf]

Histone deacetylase 1 is required for the development of the zebrafish inner ear

Yingzi He[1], Dongmei Tang[1], Wenyan Li[1], Renjie Chai[5], and Huawei Li[1,2,3,4*]

Supplemental Figure:

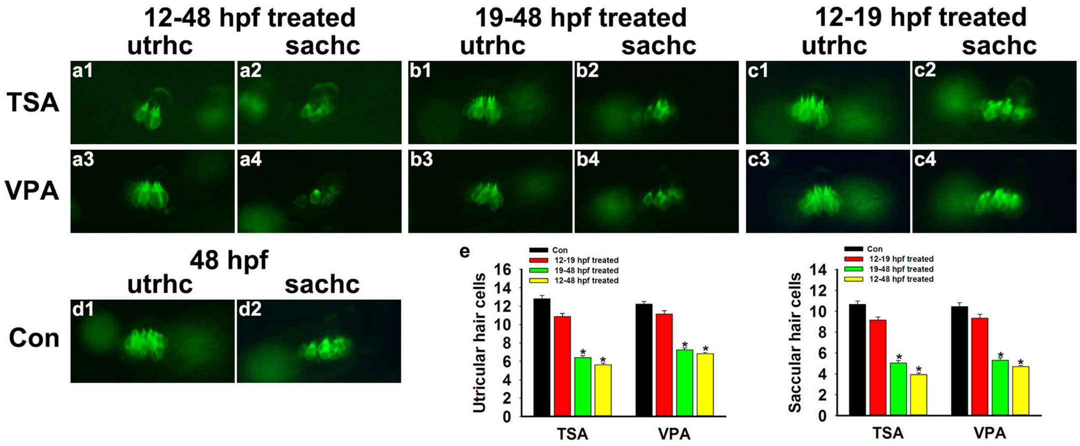

Supplemental Figure 1. Effect of HDAC inhibitor on hair cell numbers at 48 hpf. (a-c) *Brn3c*:gfp transgenic zebrafish embryos treated with HDAC inhibitors (12-48 hpf, 19-48 hpf, and 12-19 hpf) and fixed at 48 hpf to examine the numbers of hair cells in the inner ear. **(e)** Quantification of the numbers of hair cells in the inner ear for each experimental condition. Data are shown as mean ± SEM (n = 14–19). * $p < 0.05$. utrhc: utricular hair cell; sachc: saccular hair cell.

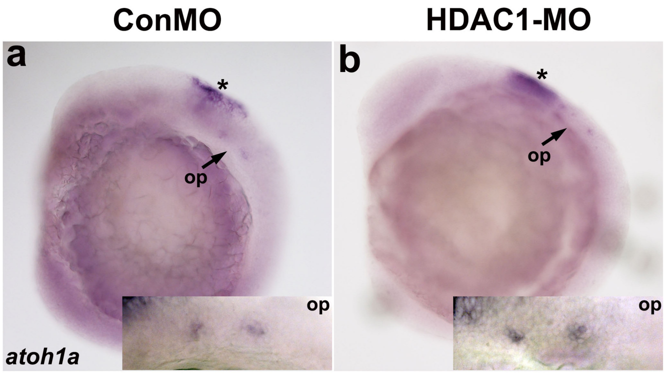

Supplemental Figure 2. Whole-mount *in situ* hybridizations to *atoh1a*. (a-b) Lateral views of embryos injected with control morpholino and HDAC1 morpholino and fixed at 14 hpf to examine expression of *atoh1a* in the otic placode. (*) dorsal hindbrain progenitors. Arrows: otic placode (op).

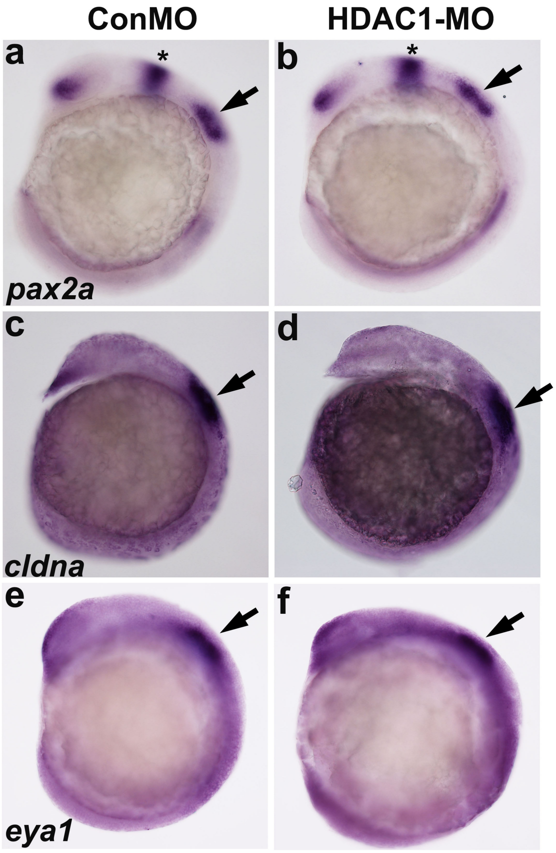

Supplemental Figure 3. Expression of otic placode markers. (a-b) Lateral views of embryos injected with control morpholino and HDAC1 morpholino and fixed at 14 hpf to examine expression of *pax2a* **(a–b)**, *cldna* **(c–d)** and *eya1* **(e–f)** in the otic placode. The otic placodes are indicated by black arrows. (*) midbrain-hindbrain boundary.
